# The nonpathogenic strain of *Fusarium oxysporum* FO12 induces Fe deficiency responses in cucumber (*Cucumis sativus* L.) plants

**DOI:** 10.1007/s00425-023-04079-2

**Published:** 2023-02-09

**Authors:** Miguel A. Aparicio, Carlos Lucena, María J. García, Francisco J. Ruiz-Castilla, Pablo Jiménez-Adrián, Manuel S. López-Berges, Pilar Prieto, Esteban Alcántara, Rafael Pérez-Vicente, José Ramos, Francisco J. Romera

**Affiliations:** 1 Departamento de Química Agrícola, Edafología y Microbiología, Edificio Severo Ochoa (C-6), Campus de Excelencia Internacional Agroalimentario de Rabanales (ceiA3), University of Córdoba, 14014 Córdoba, Spain; 2 Departamento de Agronomía, Edificio Celestino Mutis (C-4), Campus de Excelencia Internacional Agroalimentario de Rabanales (ceiA3), University of Córdoba, 14014 Córdoba, Spain; 3 Departamento de Botánica, Ecología y Fisiología Vegetal, Edificio Celestino Mutis (C-4), Campus de Excelencia Internacional Agroalimentario de Rabanales (ceiA3), University of Córdoba, 14014 Córdoba, Spain; 4 Departamento de Genética, Edificio Gregor Mendel (C-5), Campus de Excelencia Internacional Agroalimentario de Rabanales (ceiA3), University of Córdoba, 14014 Córdoba, Spain; 5 Departamento de Mejora Genética, Instituto de Agricultura Sostenible (IAS), Consejo Superior de Investigaciones Científicas (CSIC), 14004 Córdoba, Spain

**Keywords:** Biostimulant, Calcareous soil, Fe deficiency, Induced systemic resistance (ISR), Rhizospheric microorganisms

## Abstract

**Main conclusion:**

FO12 strain enhances Fe deficiency responses in cucumber plants, probably through the production of ethylene and NO in the subapical regions of the roots.

**Abstract:**

Rhizosphere microorganisms can elicit induced systemic resistance (ISR) in plants. This type of resistance involves complex mechanisms that confer protection to the plant against pathogen attack. Additionally, it has been reported by several studies that ISR and Fe deficiency responses are modulated by common pathways, involving some phytohormones and signaling molecules, like ethylene and nitric oxide (NO). The aim of this study was to determine whether the nonpathogenic strain of *Fusarium oxysporum* FO12 can induce Fe deficiency responses in cucumber (*Cucumis sativus* L.) plants. Our results demonstrate that the root inoculation of cucumber plants with the FO12 strain promotes plant growth after several days of cultivation, as well as rhizosphere acidification and enhancement of ferric reductase activity. Moreover, Fe-related genes, such as *FRO1, IRT1* and *HA1*, are upregulated at certain times after FO12 inoculation either upon Fe-deficiency or Fe-sufficient conditions. Furthermore, it has been found that this fungus colonizes root cortical tissues, promoting the upregulation of ethylene synthesis genes and NO production in the root subapical regions. To better understand the effects of the FO12 strain on field conditions, cucumber plants were inoculated and cultivated in a calcareous soil under greenhouse conditions. The results obtained show a modification of some physiological parameters in the inoculated plants, such as flowering and reduction of tissue necrosis. Overall, the results suggest that the FO12 strain could have a great potential as a Fe biofertilizer and biostimulant.

## Introduction

Iron (Fe) is one of the most abundant elements in the earth´s lithosphere. However, in calcareous soils, with pH between 7.4 and 8.5, Fe presents low solubility and availability for plants. Consequently, plants may suffer from Fe deficiency when grown in calcareous soils, showing chlorosis of the youngest leaves (Loeppert 1986; Romera et al. 2019; Taalab et al. 2019). Fe is a redox-active metal, being mainly involved in photosynthesis and mitochondrial respiration (hemoproteins involved in electron transfer) as well as protection against reactive oxygen species (Marschner 2011). In addition, it can also participate in other key processes, such as nitrogen assimilation and hormone biosynthesis (gibberellic acid, ethylene, jasmonic acid, etc.) (Hänsch and Mendel 2009).

Plants have developed two different strategies for Fe uptake from soil: Strategy I and Strategy II. Strategy II plants (Graminaceous plants) release Fe-chelating compounds, called phytosiderophores, to the rhizosphere where they chelate inorganic Fe^3+^. The resulting Fe^3+^-chelate compound is then taken up by a specific plasma membrane transporter of root epidermal cells (Mori 1999; Lucena et al. 2015). This work is devoted to Strategy I plants and consequently thereafter the descriptions are only related to this kind of plants. Strategy I plants, such as *Arabidopsis thaliana* or cucumber (*Cucumis sativus* L.), need to reduce Fe^3+^ to Fe^2+^ previous to its absorption (Brumbarova et al. 2015; Lucena et al. 2015). This reduction is mediated by a ferric reductase at the root surface, encoded by the *FRO2* gene in *Arabidopsis*. Fe^2+^ is then taken up through a Fe^2+^ transporter, encoded by the *IRT1* gene in *Arabidopsis* (Brumbarova et al. 2015; Lucena et al. 2015). When suffering from Fe deficiency, these plants induce several physiological and morphological responses in their roots, such as an enhanced ferric reductase activity, and enhanced capacity for Fe^2+^ uptake, the acidification of the rhizosphere (due to H^+^-ATPases encoded by *AHA* genes in *Arabidopsis*)*,* and an increased synthesis and release of organic acids (such as malate and citrate) and coumarins (phenolic compounds) (Romera et al. 2019). Regarding the morphological responses, it is interesting to mention the formation of subapical root hairs, cluster roots and transfer cells, intended to increase the surface of contact with soil. The enhancement of both morphological and physiological responses is significantly higher in the subapical region of the roots (Li and Lan 2017; Romera et al. 2019).

Most genes involved in physiological Fe deficiency responses are regulated by transcription factors (TFs) of the bHLH family, some of them activated by the hormone ethylene (Lucena et al. 2015). The most important and widely known TFs of this family are FIT in *A. thaliana* and FER, its homologous, in tomato (Schwarz and Bauer 2020). *FIT* is induced in roots under Fe deficiency. Another TF strongly induced in roots under Fe deficiency, and also under ISR, is *MYB72*, involved in the upregulation of some coumarin-related genes (Zamioudis et al. 2014; Romera et al. 2017).

The role of phytohormones and signaling molecules, such as ethylene and nitric oxide (NO), in the regulation of Fe deficiency responses in dicot (Strategy I) plants has been demonstrated in many studies (Lucena et al. 2006; Graziano and Lamattina 2007; García et al. 2010, 2015, 2021; Romera et al. 2017). Both ethylene (its precursor ACC) and NO can up-regulate the expression of several Fe-related genes in different Strategy I plants (Li and Lan 2017; Romera et al. 2017). Ethylene is synthetized from L-methionine, which is converted into S-adenosyl-L-methionine by the action of SAM-synthetases. Then, an ACC synthase converts this compound into 1-aminocyclopropane-1-carboxylic acid (ACC), and finally, ACC is transformed into ethylene by ACC oxidase (ACO). Ethylene is sensed by a family of receptors. When ethylene binds these receptors, activates the cleavage of the EIN2 protein, located at the endoplasmic reticulum membrane (Binder 2020). The C-terminal end of EIN2 is processed and translocated into the nucleus where it promotes ethylene signaling through different TFs, like EIN3 and EIL1 in *Arabidopsis* (Binder 2020). The EIN3 TF modulates several transcriptional cascades in which influence many genes that regulate de-etiolation or root development, among other processes (Li et al. 2017; Dolgikh et al. 2019; Houben and Van de Poel 2019). The EIN3 TF also influences the regulation of the FIT TF (Lingam et al. 2011; Yang et al. 2014).

In the past decades, it has been discovered that some nonpathogenic microorganisms of the rhizosphere can trigger ISR in plants. This phenomenon occurs by the recognition of some molecular patterns present in microorganisms, such as the bacterial flagellin or the fungal chitin. These patterns are called pathogen-(or microbe)-associated molecular patterns (PAMPs or MAMPs). When plants perceive these MAMPs or PAMPs, they activate responses that protect them against pathogen or herbivorous attack (Pieterse et al. 2014; Romera et al. 2019). Additionally, these microorganisms can modify other characteristics of plants, such as growth. This is the reason why they are called Plant Growth Promoting Bacteria or Plant Growth Promoting Rhizobacteria (PGPB or PGPR) and Plant Growth Promoting Fungi (PGPF) (Pieterse et al. 2014). Some of them, such as the ISR-eliciting ones, are also able to enhance plant Fe nutrition, probably because ISR and Fe deficiency responses share common regulators (ethylene, NO…) (Romera et al. 2019). The effects of these microorganisms on Fe nutrition are associated with their capacity to up-regulate many key Fe-related genes, like *FIT*, *MYB72*, *IRT1, FRO2,* and others (Pieterse et al. 2014; Romera et al. 2019).

It has been reported that some nonpathogenic strains of *Fusarium oxysporum* can elicit ISR (Alabouvette and Olivain 2002). In fact, the inoculation with these strains can protect against *Fusarium* wilt and other diseases, like *Verticillium*, reducing the symptoms and controlling the disease (Alabouvette and Olivain 2002; Varo et al. 2016). However, some studies have suggested that nonpathogenic strains of *Fusarium oxysporum* can elicit other special type of resistance, called endophytic-mediated resistance (EMR). This occurs when the plant acquires resistance against pathogens after being colonized by an endophytic microorganism, like *Fusarium* spp. (Constantin et al. 2020). This type of resistance differs from ISR because endophytic microorganisms typically do not confer resistance against pathogens in the aerial tissues (Lamo and Takken 2020). Additionally, it has been suggested that ethylene does not participate in EMR (Constantin et al. 2019). Nonetheless, the affirmation that the nonpathogenic strains of *Fusarium oxysporum* induce EMR is controversial since it has been demonstrated that the inoculation of *Capsicum annuum* plants with the nonpathogenic strain of *F. oxysporum* FO47 confers protection against *Verticillium dahliae* and diminishes foliar damage (Veloso and Díaz 2012).

In this work, we have inoculated cucumber plants (*Cucumis sativus* L. cv. Ashle*y*) with the nonpathogenic strain of *Fusarium oxysporum* FO12. Varo et al. (2016) already demonstrated that this strain is capable of completely inhibit *Verticillum* disease symptoms in olive trees. Based on this evidence, we postulate that the FO12 strain could behave as an ISR-eliciting microorganism, as occurs with other nonpathogenic strains of *Fusarium oxysporum* (Alabouvette and Olivain 2002). To test this possibility, we have checked whether FO12 can induce Fe deficiency responses in a dicot plant species, like cucumber, as other ISR-eliciting fungi do (Romera et al. 2019). Our results suggest that the FO12 strain can induce Fe deficiency responses in cucumber plants similarly to other ISR-eliciting microbes.

## Materials and methods

### Seed germination and plant cultivation

Seeds were surface sterilized with 20% sodium hypochlorite for 1 min and then rinsed with distilled water. Seeds were sown over the surface of wet blotting paper placed in the bottom of a tray. 20 ml of a 5 mM CaCl_2_ solution were added and seeds were covered with another layer of wet blotting paper. The tray was covered with a plastic bag and incubated at 27 °C in the dark for 2–3 days. After germination, seedlings were inserted in plastic lids and held in the holes of a thin polyurethane raft floating on aerated nutrient solution containing 10 µM Fe-EDDHA (Lucena et al. 2006). This hydroponic system was kept in the growth chamber at 22 ºC day/20 ºC night temperatures with a relative humidity of 70% and a 14-h photoperiod at an irradiance of 300 µmol m^−2^ s^−1^ for 7–8 days. The nutrient solution (without Fe) had the following composition: 2 mM Ca(NO_3_)_2_, 0.75 mM K_2_SO_4_, 0.65 mM MgSO_4_, 0.5 mM KH_2_PO_4_, 50 μM KCl, 10 μM H_3_BO_3_, 1 μM MnSO_4_, 0.5 μM CuSO_4_, 0.5 μM ZnSO_4_, 0.05 μM (NH_4_)_6_Mo_7_O_24_.

### Cultivation of fungus and inoculum preparation

FO12 strain and FO12 transformed with GFP (green fluorescent protein) were kindly provided by the ‘Patología Agroforestal’ group from the Universidad de Córdoba. FO12 strain was cultured in 250 ml PDB (Potato Dextrose Broth, Scharlau) in 1 l flasks. After 4-day-incubation at 28 ºC and 110 rpm constant shaking, the culture was filtered with a sterile nylon filter of 10 µM pore size (NYLON_10, Filtra Vibración, Barcelona, Spain) in order to separate spores from the micelia. Spores were centrifuged at 10,000 rpm during 10 min, and resuspended into 4 ml sterile distilled water. Spores were counted in a Neubauer chamber to estimate their concentration prior to inoculation. FO12 strain was cultured as described above but adding 25 ppm of hygromycin to keep selective pressure.

### Plant inoculation

**Fig. 1:**
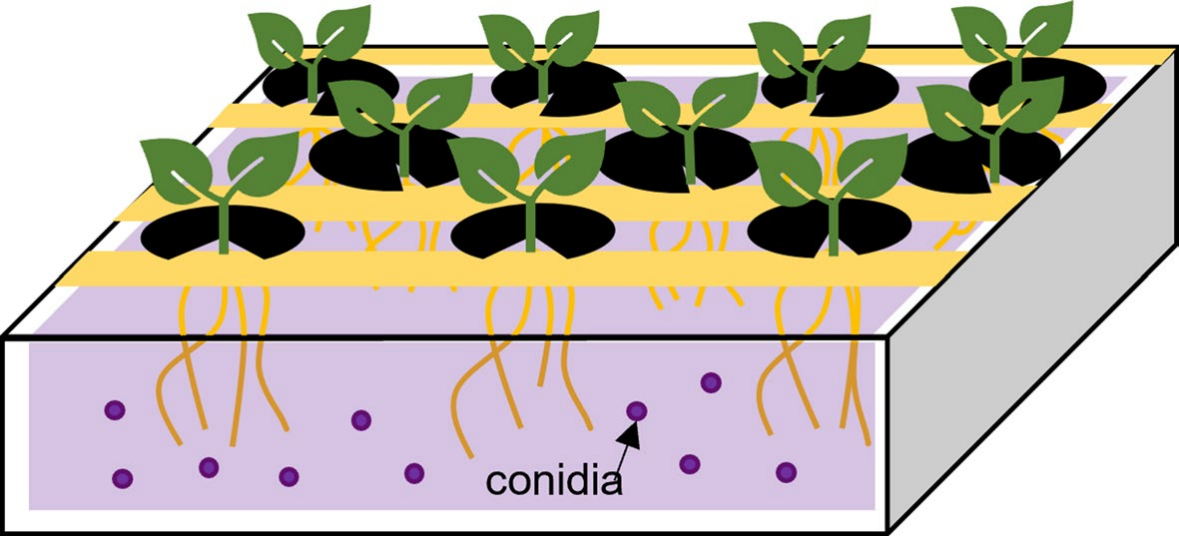
Schematic representation of the immersion inoculation method. Roots of 10-day-old plants held in plastic lids were placed in a tray containing nutrient solution and the conidia (10^7^ conidia x ml^−1^ solution) for 30 min

10-day-old cucumber seedlings were inoculated by root immersion according to Navarro-Velasco et al. (2011). First, a 10^7^ spores/ml solution was prepared with distilled water and poured in a 1.5 L capacity tray (Fig. 1). To avoid contact between solution and shoots, some lanes were covered with insulating tape. Roots were submerged in the solution during 30 min. Constant gentle shacking of the system was needed to avoid spore precipitation. After inoculation, plants were transferred to the following treatments: Fe 40 µM or -Fe. Each treatment had its corresponding control. Plants were daily harvested to determine ferric reductase activity, NO production and gene expression along time. For growth promotion determination, plants were harvested at 7 days after inoculation.

### Calcareous soil cultivation

For testing the effects of the FO12 strain in the growth of plants in a calcareous soil, plants were transferred to a calcareous soil after inoculation, and cultured under greenhouse conditions. To enhance perfusion, 2 parts of sand were mixed with 1 part of a calcareous soil. Each plant was transplanted in a 0.7 ml capacity individual pot. Plants were harvested after two months and some parameters, such as SPAD levels, flower production and leaf survival, were determined.

### Ferric reductase activity determination

Ferric reductase activity was determined as previously described (Lucena et al. 2006). In brief, roots of intact plants were submerged for 30 min in 50 ml of nutrient solution without micronutrients, pH 5.5, and then transferred to a 25 ml solution without micronutrients containing 100 mM Fe(III)–EDTA and 300 mM ferrozine, pH 5.0 (adjusted with 0.1 N KOH). Plants were incubated in this solution for 60 min under the same environmental conditions described above. The ferric reductase activity was determined spectrophotometrically by measuring the absorbance (562 nm) of the Fe (II)–ferrozine complex and using an extinction coefficient of 29,800 M^−1^ cm^−1^ (Lucena et al. 2006). After the reduction assay, roots were excised, weighed and stored at -80 ºC for later use. The results were expressed on a root fresh weight basis.

### Ferric reductase activity and acidification location

Individual roots were excised and placed over the surface of an assay solution, pH 5, gelified with 0.75% agar and supplemented with 300 mM ferrozine and 100 mM Fe(III)–EDTA for ferric reductase activity determination. For acidification determination, the nutrient solution, pH 6, was supplemented with 0.1% Purple Bromocresol (6 ml in 100 ml of nutrient solution). After 30 min incubation, pictures of the roots were taken.

### NO localization

Nitric oxide formation in the subapical region of the roots was monitored using DAF-2DA and a Zeiss AxioCam ICc 1 digital camera coupled to a stereoscopic fluorescence microscopy (Zeiss SteREO Lumar. V12 driven by Zeiss AxioVision 4.8 software; Zeiss, Jena, Germany). Images were taken with episcopic illumination at 350 ms exposition, using a GFP filter. Previously, excised roots were loaded with 5 μM DAF-2 DA in 10 mM HEPES/NaOH pH 7.5 buffer for 20 min and washed 3 times in fresh buffer.

### FO12 colonization studies in cucumber roots

The GFP-tagged FO12 transformant was used to monitor the infection and colonization process of an entire cucumber plant. More than 100 plants were inoculated with the GFP-tagged fungus, and the colonization of root tissue samples was monitored by CLSM (confocal laser scanning microscopy) over the 25 days post-inoculation period. Three different treatments were settled up in the presence of the *F. oxysporum* transformant to assess the effect of Fe concentration in the *F. oxysporum* colonization process of root tissues. Thus, 36 inoculated plants were growth in the absence of Fe, 36 in 5 µM Fe concentration, and 36 in 60 µM Fe concentration. Two plants per treatment were analyzed per day. Inoculated plants were sampled as follows: two plants per day from 1 to 10 days after inoculation (dai) (20 plants), and two plant every 2–3 days from 10 until 25 dai (16 plants). In total, all the inoculated plants were examined by CLSM.

Preparation of cucumber tissue samples for microscopic studies was done as previously described (Prieto et al. 2007), although vibratome sectioning was not necessary for CLSM analysis. Whole roots were visualized using CLSM. At least, ten different whole roots per plant were mounted in a slice with distilled water for CLSM analysis. Single confocal optical sections were collected in whole root tissues from the different treatments using an Axioskop 2 MOT microscope (Zeiss) equipped with a Krypton and an Argon laser, controlled by Zeiss Laser Scanning System LSM5PASCAL software. GFP-tagged *F. oxisporum* was exposed to 488 nm Argon laser light (detection at 500–520 nm). Data were recorded and the images transferred for analysis to Zeiss LSM Image Browser version 4.0 (Zeiss). Confocal stacks were mounted and analyzed to assess colonization of *F. oxisporum*. Images included in Figs. 8 and 9 were generated from projections of adjacent confocal optical sections). Final figures were processed with Photoshop 10.0 software (Adobe Systems, San Jose, CA, USA).

### q-RT-PCR analysis

Roots were ground to a fine powder with a mortar and pestle in liquid nitrogen. Total RNA was extracted using Tri Reagent solution (Molecular Research Center, Inc. Cincinnati, OH, USA) according to the manufacturer's instructions. M-MLV reverse transcriptase (Promega, Madison, WI, USA) was used to generate cDNA from 3 μg of DNase-treated root RNA, using random hexamers for amplification. The study of gene expression by qRT-PCR was performed by using a qRT-PCR Bio-Rad CFX connect thermal cycler. The amplification profile consisted in cycles with the following conditions: initial denaturation and polymerase activation (95 °C for 3 min), amplification, and quantification (90 °C for 10 s, 57 °C for 15 s, and 72 °C for 30 s), and a final melting curve stage of 65 to 95 °C with increment of 0.5 °C for 5 s, to ensure the absence of primer dimer or nonspecific amplification products. PCR reactions were set up in 20 μl of SYBR Green Bio-RAD PCR Master Mix, following the manufacturer's instructions. Controls containing water instead of cDNA were included to detect contamination in the reaction components. Normalization was performed using two reference genes (*CsActin* and *CsCylco*). The primers used are listed in Table 1.

**Table 1:** Primer pairs for cucumber (*Cucumis sativus*)

Gene	Sequence (5′–3′)
*CsFRO1* (AY590765)	F-ATACGGCCCTGTTTCCACTT
	R-GGGTTTTGTTGTGGTGGGAA
*CsIRT1* (AY590764)	F-GCAGGTATCATTCTCGCCAC
	R-ATCATAGCAACGAAGCCCGA
*CsHA1* (AJ703810)	F-GGGATGGGCTGGTGTAGTTTG
	R-TTCTTGGTCGTAAAGGCGGT
*CsACO1* (FN544066)	F-TTTGGTGGCGGAGGAGAAAA
	R-ATGGCTTCAAACCTCGGCTC
*CsACO3* (AF033583)	F-ACTCAAAACAGTGGAACTGGA
	R-GGGGTACACTTCCTTCTTCTCC
*CsEIN2* (KF245636)	F-TGCCGACAAGGTTAAATGGG
	R-TGCTGCTGCACAATAGAAGA
*CsEIN3* (KF245636)	F-GCTTTCTGGGGTTGCGATTT
	R-CCGAACAGTCTCCCAAAGCA
CsActin* (XM_004136807)	F-AACCCAAAGGCAAACAGGGA
	R-TCCGACCACTGGCATAGAGA
CsCyclo* (NM_001280769)	F-ATTTCCTATTTGCGTGTGTTGTT
	R-GTAGCATAAACCATGACCCATAATA

*Reference genes

### Statistical analysis

All statistical analyses were performed using GraphPad Prism 8. To determine if the variables studied follow a normal distribution, the Shapiro–Wilk normality test was applied. If the signification value was greather than 0.05, the data were considered parametric. On the other hand, if the signification value was below 0.05, the data were considered as non-parametric. Student´s *t-*test (parametric) or Mann–Whitney test (non-parametric) were used to determine significant differences (*P* < 0.05) between inoculated and control treatments.

Analysis of variance (One-way ANOVA) and Dunnett´s test were used to compare gene expression of control treatments and inoculated treatments at different times, setting the threshold fot statistical significance at *P* < 0.05.

## Results

Following are the results obtained in this work showing the effects of FO12 inoculation of cucumber plants on growth, Fe deficiency responses (ferric reductase activity and acidification), NO production and gene expression. Results describing the colonization of the roots by the GFP-tagged FO12 and those showing the effects of FO12 on the growth of cucumber plants in a calcareous soil are also presented.

### Effect of the inoculation with the FO12 strain on growth promotion

**Fig. 2:**
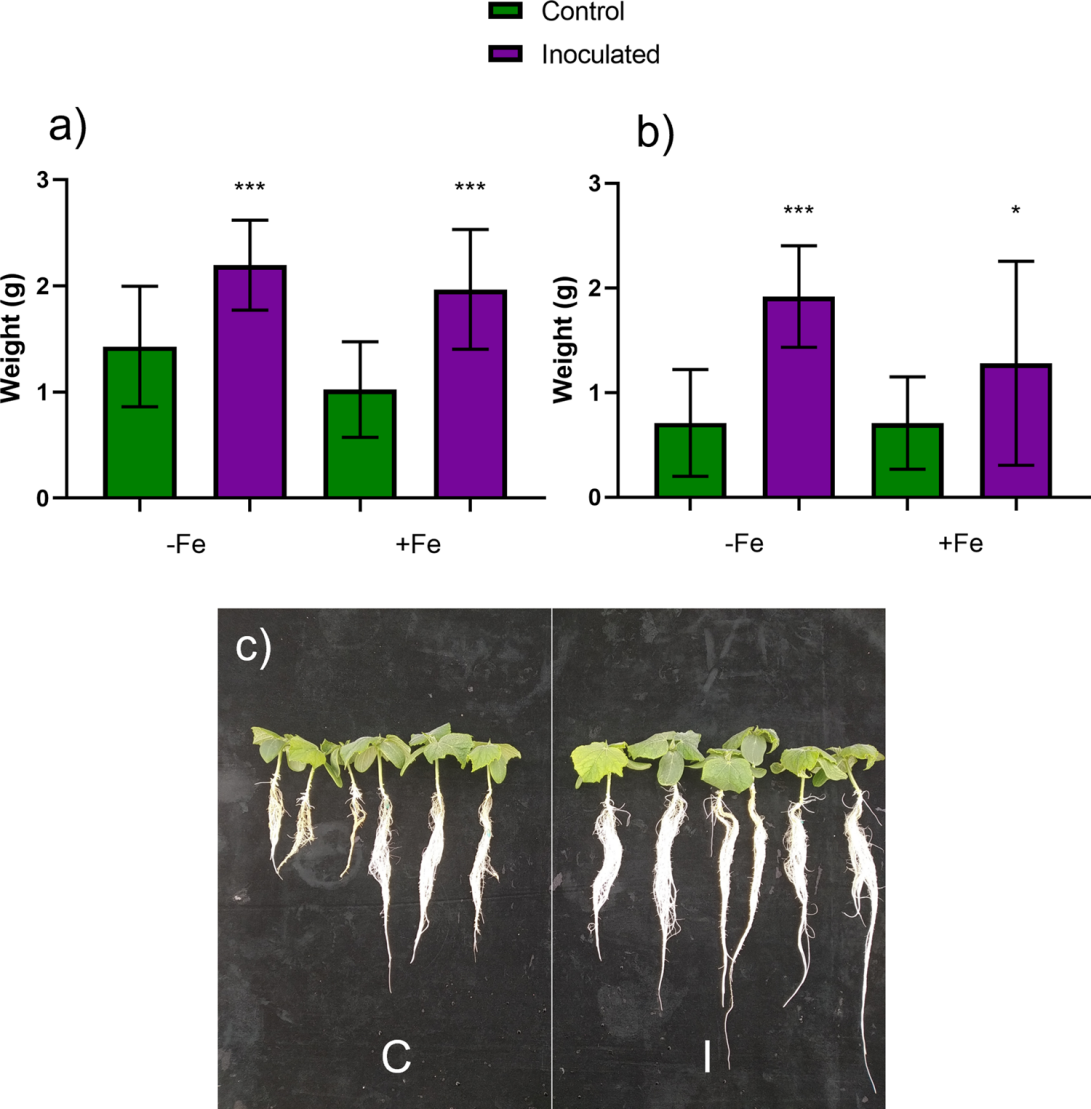
Growth promotion of cucumber plants inoculated with the FO12 strain. To determine growth promotion, half of the 10-day-old plants were inoculated, and then Inoculated and Control plants cultivated for 7 additional days either under Fe deficiency (– Fe) or Fe 40 µM (+ Fe). After that time, roots and shoots were excised and weighed separately. **a** Shoot fresh weight. **b** Root fresh weight. **c** Comparison between Control and Inoculated plants of the -Fe treatment. C, Control (green); I, Inoculated (violet). Within each Fe treatment, *P < 0.05 or ***P < 0.001 indicate significant differences in relation to the Control treatment (values are the means ± SE of 30 replicates)

To determine growth promotion, half of the 10-day-old cucumber plants were inoculated with the FO12 strain, and then inoculated and control plants cultivated for 7 additional days either under Fe deficiency (– Fe) or Fe 40 µM (+ Fe). After 7 days of the inoculation with the FO12 strain, there was a significant growth promoting effect both in shoots and roots either under Fe deficiency or Fe sufficiency (Fig. 2).

### Effect of the inoculation with the FO12 strain on the induction of Fe deficiency responses (ferric reductase activity and acidification)

**Fig. 3:**
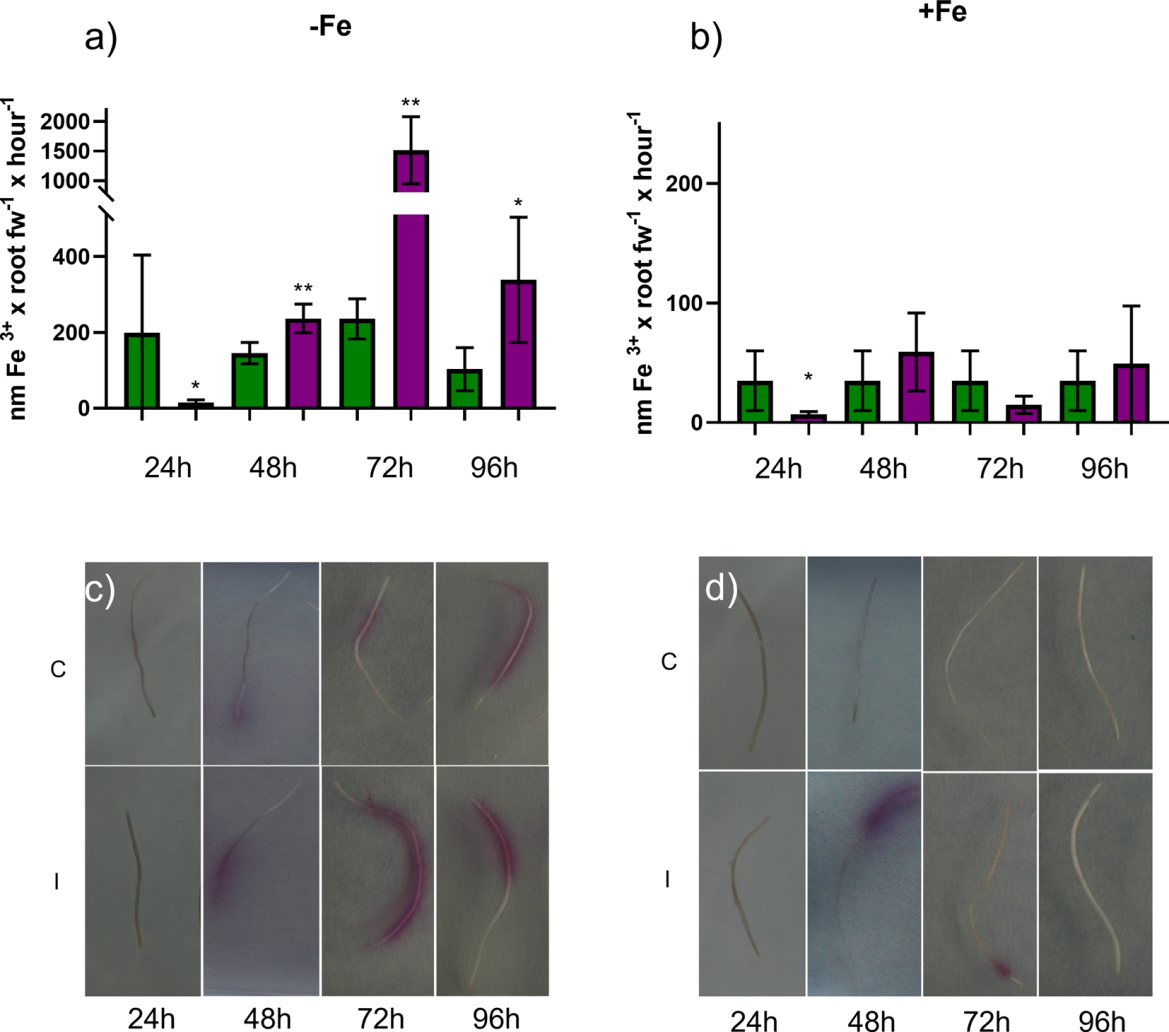
Time course of ferric reductase activity (FRA) in inoculated and non-inoculated cucumber plants. After inoculation, plants were transferred to nutrient solution with either Fe 40 µM (+ Fe) or without Fe ( – Fe) for 24, 48, 72 and 96 h. **a** FRA under Fe deficiency. **b** FRA under Fe sufficiency. **c** FRA location on Fe-deficient roots. d FRA location on Fe-sufficient roots. C, Control (green); I, Inoculated (violet). Within each time, *P < 0.05, **P < 0.01 or ***P < 0.001 indicate significant differences in relation to the Control treatment (values are the means ± SE of six replicates)

To study the effect of FO12 strain on ferric reductase activity and acidification, after inoculation, cucumber plants grown in hydroponic system were transferred to nutrient solution with either Fe 40 µM (+ Fe) or without Fe ( – Fe) for 24, 48, 72 and 96 h. After 24 h of the inoculation, there was a sharp inhibition of ferric reductase activity (FRA) either under Fe deficiency or Fe sufficiency (Fig. 3). However, later on, there was an enhancement of FRA along time in the Fe-deficient inoculated plants, reaching its maximum at 72 h after inoculation (Fig. 3a). In Fe-sufficient inoculated plants, there was a slight induction of FRA most of the days, in contrast to control plants, but the differences were not significant (Fig. 3b). Similar results were observed on FRA location on agar, being the induction mainly located in the subapical region of the roots (Fig. 3c, d).

**Fig. 4:**
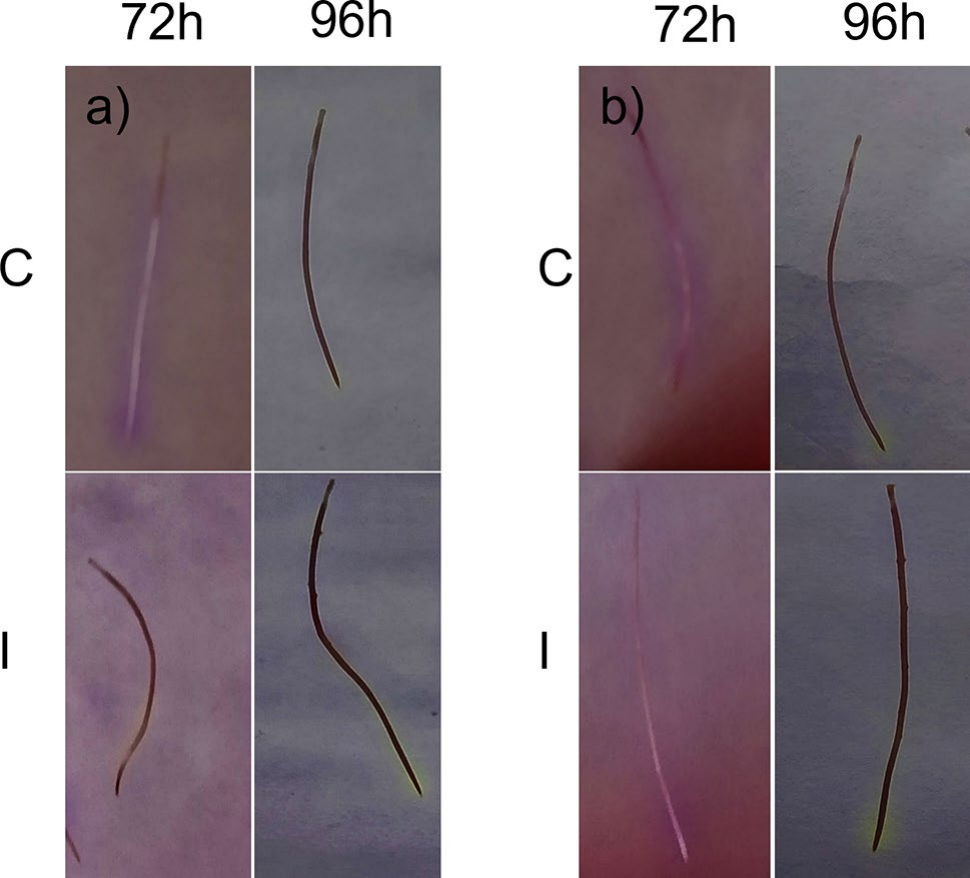
Effect of FO12 strain on the acidification response. Plants were cultivated as described in Fig. 3. The acidification response was determined placing excised roots on agar supplemented with 0.1% purple bromocresol. **a** Fe deficiency. **b** Fe sufficiency

In relation to the acidification response, it is widely known that dicot plants enhance H^+^ release to the rhizosphere in response to Fe deficiency, due to the action of H^+^-ATPases (Lucena et al. 2015). Some ISR-eliciting microorganisms can enhance this response (Romera et al. 2019). In order to assess H^+^ production, isolated roots were submerged on agar plates containing nutrient solution without Fe and Bromocresol Purple (pH indicator). The acidification response can be identified by a yellowing area around root surface, frequently located closer to the subapical region. As shown in Fig. 4, the acidification was clearly higher in the inoculated roots, mainly under Fe deficiency, where the effect could be seen after 72 h of the inoculation. At 24 and 48 h, there was no acidification effect in either treatment (not shown).

### Effect of the FO12 strain on the expression of Fe-related genes

**Fig. 5:**
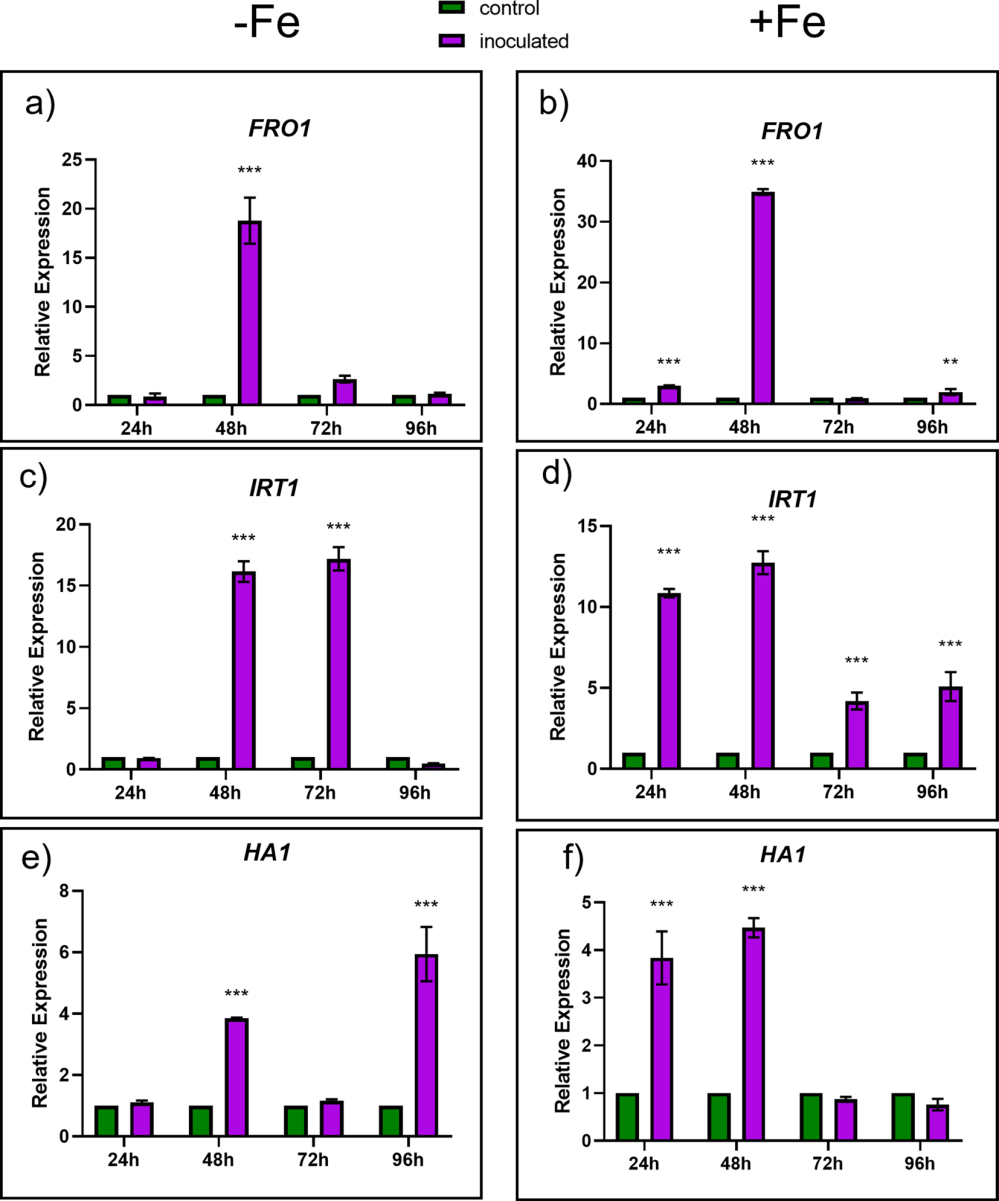
Effect of the inoculation with the FO12 strain on the expression of the Fe-related genes *FRO1, IRT1* and *HA1* under Fe deficiency (left) or Fe sufficiency (right). Half of 10-day-old plants were inoculated while others not, and then transferred to nutrient solution containing either 40 µM Fe (+ Fe) or no Fe (– Fe). Relative expression was calculated in relation to the control (+ Fe) of each day. Within each time, *P < 0.05, **P < 0.01 or ***P < 0.001 indicate significant differences in relation to the Control treatment

To look further in the induction of Fe deficiency responses by the FO12 strain, we then analyze the expression of several genes associated with these responses in cucumber: *FRO1* (ferric reductase), *IRT1* (Fe^2+^ transporter) and *HA1* (H^+^-ATPase) (Waters et al. 2007). As shown in Fig. 5, the expression of all these Fe-related genes was clearly and transitorily enhanced by the inoculation with FO12 either under Fe deficiency or Fe sufficiency. Depending on the genes and Fe condition, the maximum expression was attained at 48 h or 96 h (Fig. 5).

### Effect of the FO12 strain on the expression of ethylene-related genes

**Fig. 6:**
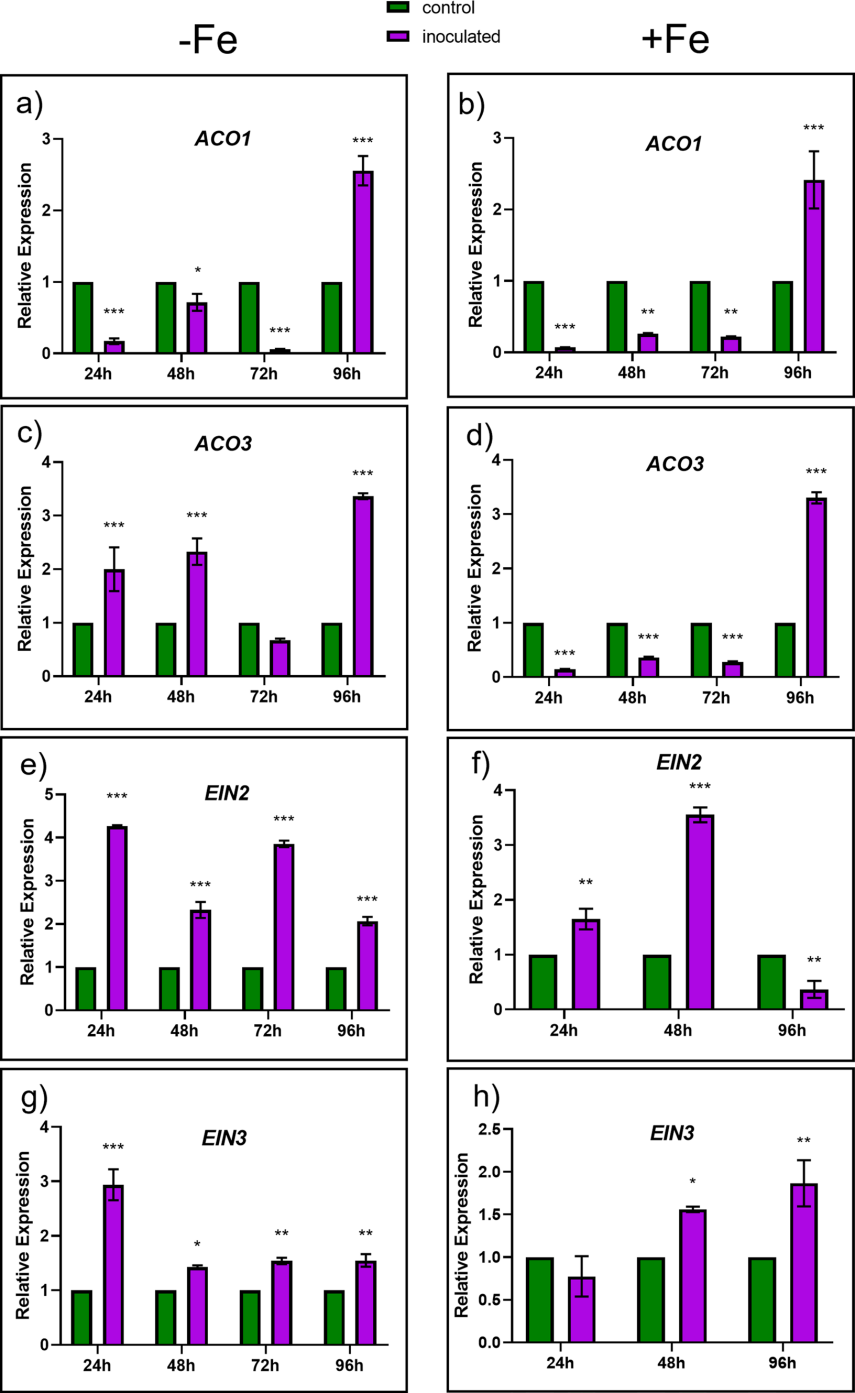
Effect of the inoculation with the FO12 strain on the expression of the ethylene-related genes *ACO1, ACO3, EIN2* and *EIN3* under Fe deficiency (left) or Fe sufficiency (right). Treatments and conditions as in Fig. 7. Relative expression was calculated in relation to the control of each day. Within each time, *P < 0.05, **P < 0.01 or ***P < 0.001 indicate significant differences in relation to the Control treatment

Besides Fe-related genes, we also analyze the expression of ethylene-related genes since this hormone has been implicated, along with NO, in the activation of several Fe deficiency responses (García et al. 2010, 2011; Lucena et al. 2015). To assess the possible effect of FO12 on the expression of ethylene-related genes, we analyze the genes *ACO1* and *ACO2*, encoding ACC oxidases, and *EIN2* and *EIN3*, related to the ethylene signaling pathway (García et al. 2021). As previously shown with the Fe-related genes, the expression of all the ethylene-related genes was clearly and transitorily enhanced by the inoculation with FO12 either under Fe deficiency or Fe sufficiency (Fig. 6). In Fe-deficient plants, FO12 induced most of the genes after 24 h of the inoculation while in Fe-sufficient plants the induction was generally delayed in relation to the Fe-deficient ones (Fig. 6).

### Effect of the inoculation with the FO12 strain on nitric oxide (NO) production

Nitric oxide (NO) production increases in Fe-deficient roots, where it has been involved in the induction of several Fe deficiency responses (Graziano and Lamattina 2007; García et al. 2010, 2011). To assess whether FO12 influences its production, NO formation was determined by incubating excised roots with DAF-2DA, a molecule that specifically reacts with NO, producing triazolofluorescein, a compound that emits fluorescence when is excited at 491 nm.

**Fig. 7:**
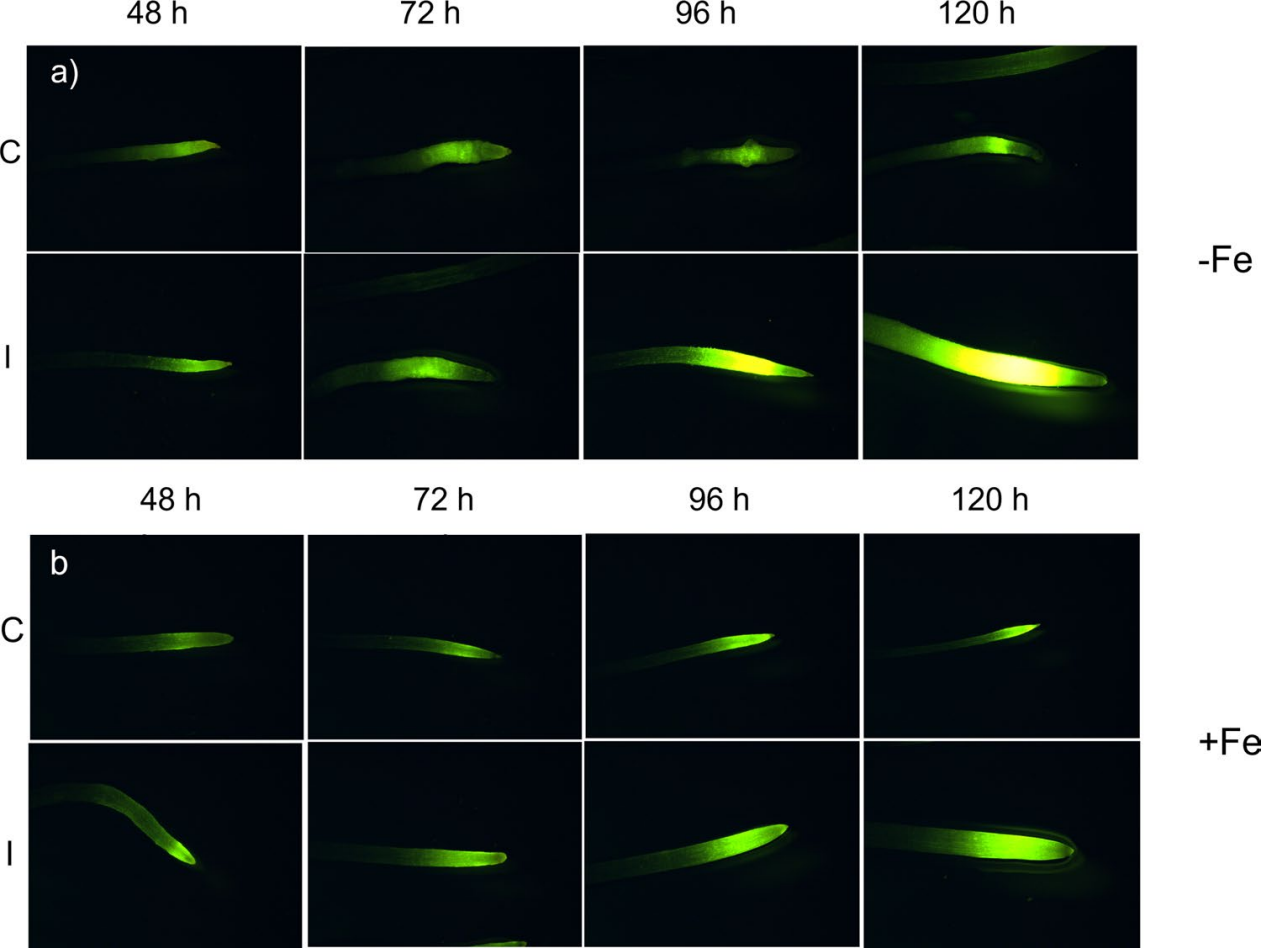
Effect of the inoculation with the FO12 strain on NO production in cucumber roots. Half of 10-day-old plants were inoculated while others not, and then transferred to nutrient solution containing either 40 µM Fe (+ Fe; bottom) or no Fe ( – Fe; top). Root samples were taken at 48, 72, 96 and 120 h. NO was visualized with the DAF-2DA fluorescent dye. Notice root swollen in the inoculated treatment at 96 h and 120 h. **b** Fe sufficiency

Inoculated plants cultured on Fe deficiency manifested a progressive increment of NO production along time when compared with control treatments, reaching the maximum at 120 h (Fig. 7). NO production was mainly located in the subapical region of the roots (Fig. 7). Furthermore, at 96 and 120 h, there was an increase of root swollen in the inoculated treatment (Fig. 7). A similar pattern was observed upon Fe-sufficient conditions regarding the evolution along time (Fig. 7). NO production increased progressively in inoculated plants from 24 to 120 h, although to lower levels than under Fe deficiency. Presence of root swollen was not observed in any of the Fe-sufficient treatments (Fig. 7).

### FO12 colonization of cucumber root tissues

**Fig. 8:**
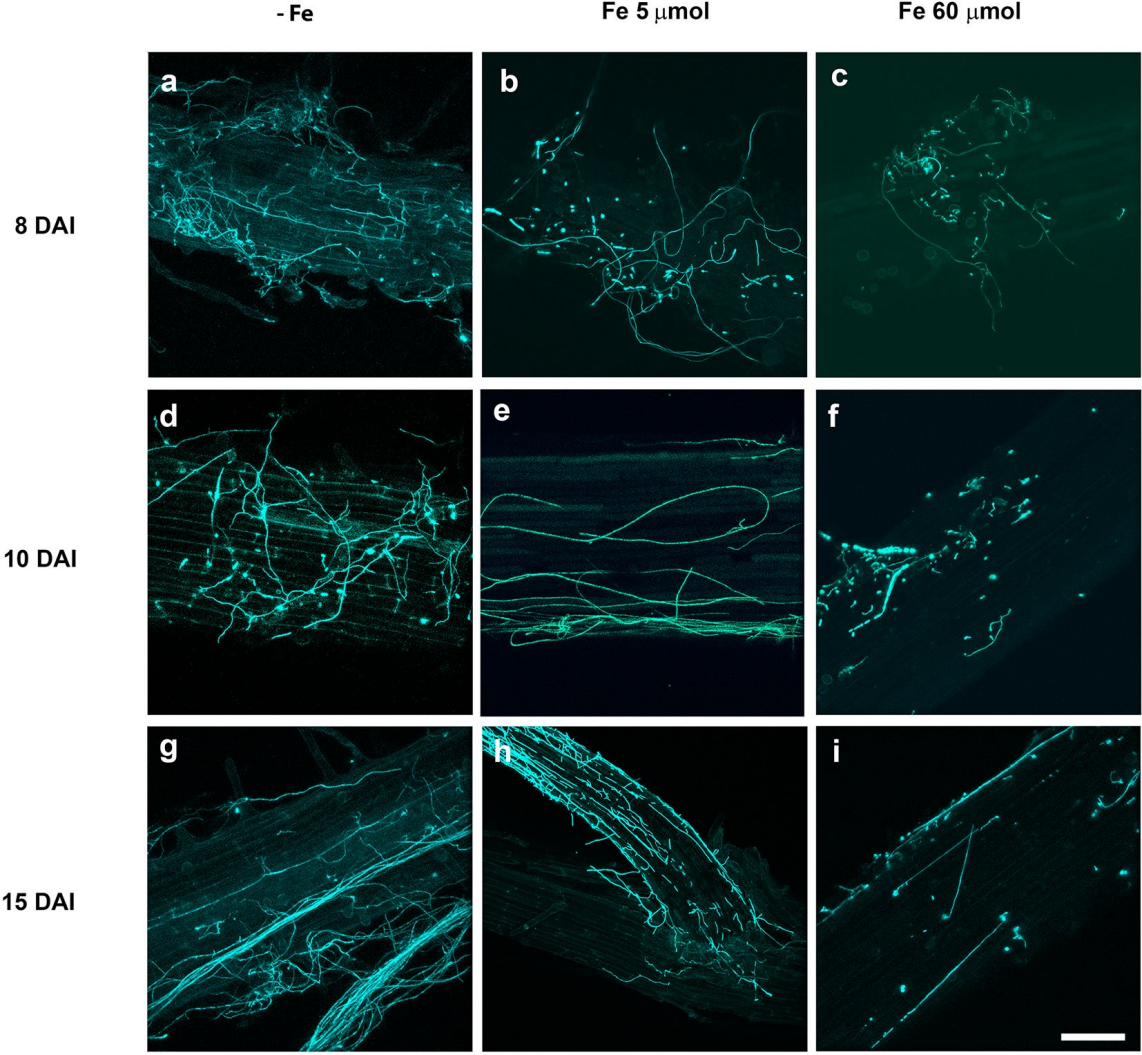
CLSM images of the time-course colonization processes of cucumber roots by the FO12-GFP-tagged strain. Confocal analysis was carried out on 4–5 cm long roots to show surface FO12 strain colonization. Images are projections of 20 adjacent confocal optical sections. The focal step between confocal optical sections was 0.5 μm. Surface colonization by FO12 on roots of cucumber plants growing without Fe (**a**, **d**, **g**), with a supplement of 5 μM Fe (**b**, **e**, **h**) or 60 μM Fe (**c,** **f**, **i**), at 8 dai (**a**–**c**), 10 dai (**d**–**f**) and 15 dai (**g**–**i**). Most of the conidia had germinated and proliferation of hyphae could be observed on the root surface of plants growing with no Fe or 5 μM Fe (**a**, **d**, **g**, **b**, **e**, **h**). Proliferation of hyphae in roots of plants growing with 60 μM Fe (**c**, **f**, **i**) was clearly lower than in those growing with no Fe (**a**, **d**, **g**) or 5 μM Fe (**b**, **e**, **h**). Scale bar represents 50 μm in all panels

**Fig. 9:**
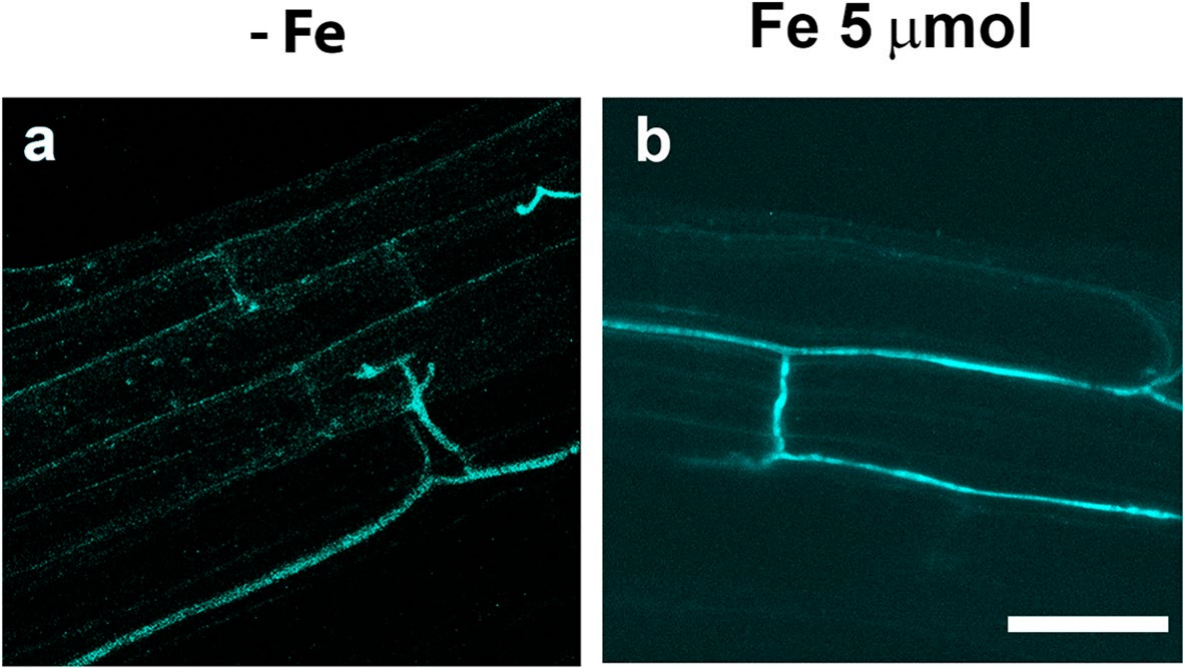
CLSM of the colonization of cucumber roots by the GFP-tagged strain of FO12 (in green). Confocal analysis was carried out on 4–5 cm long roots to show inner FO12 colonization. Images are projections of 10 adjacent confocal optical sections. The focal step between confocal optical sections was 1 μm. **a** Internal colonization of cucumber root tissues by FO12 in plants growth with no Fe. **b** Internal colonization of cucumber root tissues by FO12 in plants growth with 5 μM Fe. Scale bar represents 10 μm in both panels

Colonization of cucumber root tissues by GFP-tagged FO12 was visualized using CLSM in the absence of Fe and compared with the colonization process observed in the presence of different concentrations of Fe (5 and 60 µM). We found that the combined use of CLSM and the GFP-tagged FO12 derivative enabled the in situ localization of the fungus on/in cucumber roots without any tissue manipulation, as previously reported for *Verticillium dahliae* in olive roots (Prieto et al. 2009). CLSM imagery showed that cucumber roots were slightly colonized by conidia of FO12 2 dai in the three different treatments (data not shown). Conidia did germinate similarly over cucumber roots in the three different treatments (no Fe, 5 and 60 µM Fe). Clear differences were observed in FO12 colonization progress on cucumber roots at 8 dai, when the colonization of cucumber root surface by FO12 was more profuse in the absence of Fe, or in the presence of low concentration of Fe (5 µM), than in the presence of high concentration of Fe (60 µM) (Figs. 8, 9). In fact, FO12 conidia germination seemed to fail in the root surface of plants growing with a 60 µM Fe concentration, as very few hyphae were detected even after 15 dai (Fig. 9).

We continued analyzing plants by CLSM until 25 dai although no significant changes in sampled plants were observed from 10 dai until the end of the experiment. It is worthy to mention that the colonization progress of FO12 on the cucumber root surface was not uniform in any of the treatments studied. This mean that some root regions showed an abundance of hyphal colonization while others were completely devoid of the fungus. Internal colonization studies of cucumber roots by FO12 were also carried out using CLSM. We found that the cortical tissue of cucumber roots was internally colonized by FO12 at early stages (6 dai) when plants were growing with no or low concentration of Fe (Fig. 9a, b). However, although internal colonization of the intercellular spaces of the cortical cells was detected in roots of plants growing with no or low concentration of Fe, FO12 hyphae were not observed proliferating in the vascular tissue at any time. In contrast, FO12 was only detected on the cucumber root surface and never within the interior of the cortical tissue, up to the end of the experiment in roots of cucumber plants growing in 60 µM Fe concentration.

### Effects of the inoculation with the FO12 strain on the growth of cucumber plants cultured in a calcareous soil

**Fig. 10:**
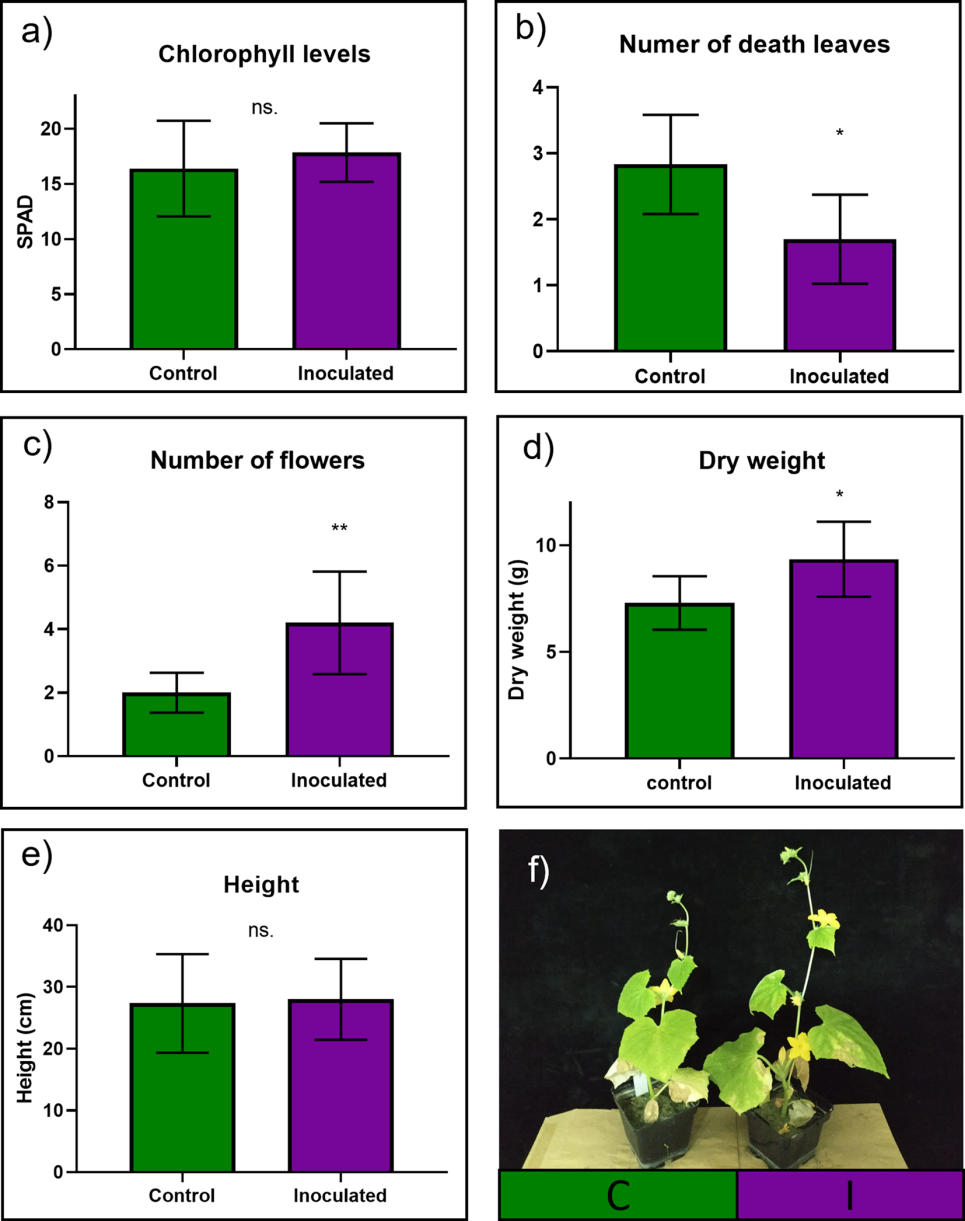
Effect of the FO12 strain on the growth of cucumber plants cultured on calcareous soil. Plants were cultured as described in Fig. 2 for 10 days and then transplanted to pots of 0.7 L capacity containing calcareous soil. Plants were harvested after 2 months. C, Control; I, Inoculated. *P < 0.05 or **P < 0.01 indicate significant differences in relation to the Control treatment (values are the means ± SE of ten replicates)

In order to identify the effects of the FO12 strain at alkaline pH, cucumber plants were cultivated in pots with a calcareous soil under greenhouse conditions. To assess these effects, some parameters, such as chlorophyll levels, leaf survival, flowering, height, and dry weight, were determined. To determine chlorophyll levels, the SPAD method was used. The SPAD determinations did not manifest the presence of significant differences between control and inoculated treatments (Fig. 10a). To determine leaf survival, the number of dead leaves was counted. Clearly, inoculated plants manifested a significant lower number of dead leaves than control plants (Fig. 10b, f). Furthermore, to assess flower production, the number of flowers per plant was determined. In this case, the inoculated plats manifested a very significant higher number of flowers than control plants (Fig. 10c, f). Finally, to determine growth promotion, shoot height and dry weight were measured. Even though there were not significant differences between control and inoculated plants regarding height, dry weight was significantly higher in inoculated plants (Fig. 10d, e, f).

## Discussion

Dicot (Strategy I) plants enhance the acquisition of Fe from soil by a complex set of physiological and morphological responses in their roots, like the induction of a ferric reductase, the enhancement of proton excretion in the rhizosphere, the induction of a Fe^2+^ transporter in the subapical region of the roots and the formation of root hairs (Brumbarova et al. 2015; Lucena et al. 2015). Evidence suggests that the exposure of dicot plants to Fe deficiency leads to an increase of ethylene production in their roots (Romera et al. 1999), which has been implicated in the induction of most Fe deficiency responses (Romera and Alcántara 2004; Lucena et al. 2015; Li and Lan 2017; Romera et al. 2017). As far as is already known, ethylene acts through the induction of the FIT TF and other bHLH TFs (Lucena et al. 2006, 2015; Yang et al. 2014; Li and Lan 2017). It has also been demonstrated that ethylene can interact with NO and other phytohormones, such as auxin. In fact, NO and ethylene are produced in the subapical region of the roots, where they also promote the formation of subapical root hairs (Graziano and Lamattina 2007).

Several works have demonstrated that the application of some ISR-eliciting microorganisms to plants can enhance their Fe acquisition from soil through the induction of Fe deficiency responses (Zamioudis et al. 2015; Zhou et al. 2016; Romera et al. 2019). ISR elicited by rhizosphere microorganisms is regulated by ethylene in plants (Pieterse et al. 2014). In addition, NO production in roots can increase in inoculated plants with some ISR-eliciting microorganisms (Creus et al. 2005; Zamioudis et al. 2015; Chang et al. 2021). Considering the key role of these phytohormones and signaling molecules in both ISR and Fe deficiency responses, a crosstalk between them has been suggested (Romera et al. 2019). Probably, ISR-eliciting microorganisms can induce Fe deficiency responses in dicot plants through the increased production of ethylene and NO (Romera et al. 2019).

Several studies have considered that some nonpathogenic strains of *F. oxysporum* can elicit ISR, protecting the plant against the attack of pathogens (Alabouvette and Olivain 2002). CS-20 and FO47 strains are the most widely studied of this group (Larkin and Fravel 1999; Pantelides et al. 2009; Validov et al. 2011; Veloso and Díaz 2012). In addition, some authors have reported other probable ISR-eliciting strains, such as F2 and FO12 (Pantelides et al. 2009; Mulero-Aparicio et al. 2019). According to Constantin et al. (2019), endophytic microorganisms, like *Fusarium spp*., can elicit EMR, a type of resistance independent of ethylene, in contrast to ISR. Consequently, these authors have suggested that the colonization of plants by nonpathogenic strains of *F. oxysporum* induce resistance in an ethylene-independent manner.

The aim of the present work has been to determine whether the FO12 strain can induce Fe deficiency responses in a dicot plant species, like cucumber, and whether it induces them in an ISR or EMR dependent manner. If this fungus can promote Fe deficiency responses in plants, then it could be considered as a potential biofertilizer. Our results show that the FO12 strain induces Fe deficiency responses, such as an enhancement of ferric reductase activity, rhizosphere acidification and formation of swollen root tips (Figs. 3, 4, 7). In a similar way, Martínez-Medina et al. (2017) demonstrated that *Trichoderma spp.* induced ferric reductase activity in *A. thaliana* and tomato plants. The expression of Fe-related genes, such as *CsFRO1, CsIRT1* and *CsHA1*, was also enhanced by the action of the FO12 strain (Fig. 5). Zamioudis et al. (2014, 2015) also reported that the root inoculation of *Arabidopsis thaliana* with the WCS417 strain of *Pseudomonas simiae* upregulates the expression of *MYB72* and other Fe acquisition genes, such as *FRO2* and *IRT1.* Similarly, Zhou et al. (2016) demonstrated that the inoculation of *A. thaliana* plants with *Paenibacillus polymyxa* BFKC01 strain promotes growth and enhances the expression of *FRO2, FIT* and *IRT1*. Martinez-Medina et al. (2017) also demonstrated that tomato plants exposed to *Trichoderma* volatiles were able to induce Fe deficiency genes, such as *LeFRO, LeIRT* and *LeFER*.

The way FO12 induces Fe deficiency responses is not known but our results suggest that it could induce them by affecting ethylene and NO production as other ISR-eliciting microorganisms do (Romera et al. 2019). In fact, our results show an upregulation of ethylene synthesis and signaling genes upon the inoculation with FO12 (Fig. 6). In addition to ethylene, we have also found that the inoculation of cucumber plants with the FO12 strain enhances the production of NO in the subapical region on the roots (Fig. 7). This increment of NO was coupled with the formation of root hairs and swelling in the root subapical region. García et al. (2011) also noticed the formation of root swelling coupled to the increment of NO when cucumber plants were cultured under Fe deficiency.

*Fusarium oxysporum* is an endophytic microorganism, which means that this microbe can grow inside the plant. According to literature, this microorganism is not able to colonize the vascular system of the plant. Instead, it forms fungal hyphae along the root cortex and the endodermis (Constantin et al. 2020). Our results show that the FO12 strain colonizes the intercellular spaces of the cortical cells (Fig. 8), further supporting this evidence. The inoculation of cucumber plants with the FO12 strain is facilitated by the depletion of Fe from the nutrient solution. On the other hand, a higher concentration of Fe interferes with root colonization (Figs. 8, 9). These results agree with those obtained by Guerra and Anderson (1985) showing that *Phaseolus vulgaris* plants growing in a hydroponic system with restricted Fe and B were more susceptible to infection by *Fusarium* wilt. Taken together, all these results indicate that Fe depletion makes the plant more susceptible to pathogen attack by the pathogenic *Fusarium* races and to endophytic colonization by the nonpathogenic ones, such as FO12.

According to Constantin et al. (2019), endophytic microorganisms induce EMR. The authors suggested that this type of resistance is independent of ethylene, in contrast to ISR. This statement mean that the colonization of the plant by nonpathogenic strains of *F. oxysporum* would induce resistance in an ethylene-independent manner. However, our results show that the expression of ethylene-related genes was clearly enhanced at certain times upon inoculation with the FO12 strain (Fig. 6). Furthermore, NO levels were also increased with the inoculation in the subapical region of the roots (Fig. 7). Kavroulakis et al. (2007) demonstrated that ethylene deficient mutants of tomato inoculated with *F. solani* were more susceptible to pathogen attack, supporting a role for ethylene in the acquisition of resistance against *Fusarium*. Furthermore, NO and ethylene enhance the expression of several Fe acquisition genes in *Arabidopsis* (García et al. 2010, 2011)*.* Considering that FO12 causes upregulation of ethylene-related genes (Fig. 6) and increases NO production (Fig. 7), it would be possible that FO12 could induce Fe deficiency responses in an ethylene/NO-dependent manner. This mean that the FO12 strain would induce ISR and not EMR. However, additional experiments with ethylene inhibitors and NO scavengers would be necessary to confirm this hypothesis. Our results also show that the effects of the FO12 strain is less potent when plants are growing under Fe sufficiency conditions (Figs. 3, 4, 7). Romera et al. (2019) also indicated that the induction of Fe acquisition genes by ethylene and/or NO is much lower when the plants are growing in the presence of high levels of Fe. Several authors also reported that ISR-eliciting microbes have less effect on the induction of Fe deficiency responses when plants are growing in presence of Fe. Zamioudis et al. (2015) reported that *P. simiae* WCS417 can induce Fe-related genes, such as *MYB72* and *FRO2*, in *A. thaliana* either under Fe-deficiency or Fe-sufficient conditions, but this induction is much lower when plants are growing in the presence of high levels of Fe. Similarly, Zhou et al. (2016) demonstrated that *P. polymyxa* BFKC01can enhance the induction of *FIT*, *IRT1* and *FRO2*, as well as ferric reductase activity, in *A. thaliana* plants independently of the Fe concentration, but this induction decreased when the plants were cultivated under Fe-sufficient conditions. Returning to our results, we found that inoculation with FO12 strain of Fe-sufficient cucumber plants upregulated *FRO1* gene expression (48 h) (Fig. 5) but the enzymatic activity was not activated (48 h) (Fig. 3). Usually, there is a one-way correlation between gene overexpression and the induction of the enzymatic activity, although both may sometimes be subject to post-transcriptional regulation (Connolly et al. 2003).

Besides the induction of Fe deficiency responses, FO12 also promotes growth (Fig. 2) as other PGPF and PGPB do (Pieterse et al. 2014). Several authors have reported similar results with other beneficial rhizosphere microorganisms. For example, Bakhshandeh et al (2020) used a combination of four microorganisms to inoculate soybean seeds and this produced a clear growth promoting effect on the plants. Fontenelle et al. (2011) also reported a highly significant growth promoting effect using several isolates of *Trichoderma spp.* to inoculate tomato plants under greenhouse conditions.

Several authors have shown that the application of PGPR or PGPF is an efficient method to ameliorate Fe chlorosis in calcareous soils (Ipek et al. 2014; Liu et al. 2019; El Komy et al. 2020). Ipek et al. (2014) were able to promote growth and enhance mineral uptake by inoculating strawberry plants with some isolates, such as *Agrobacterium, Bacillus* and *Alcaligenes*. In the same way, Liu et al. (2019) were able to promote growth when inoculating alfalfa plants with *Pseudomonas aeruginosa* and *Enterobacter aerogenes* cultured under saline-alkali conditions in greenhouse. In addition, El Komy et al. (2020) ameliorated the symptoms produced by *Fusarium solani, Macrophomina phaseolina* and *Rhizoctonia solani* on sunflower plants cultured in a calcareous soil under field conditions, and produced an evident growth promoting effect with the inoculation of a mixture of rhizobacteria. Similarly, our results showed that the inoculation of cucumber plants with the FO12 strain can ameliorate the deleterious effects caused by alkaline pH on cucumber plants. Another physiological parameter that has been proved to be influenced by rhizosphere microorganisms is the timing of flowering. According to Lu et al. (2018), the root microbiota can modulate flowering through IAA (auxins), delaying the flowering process in *A. thaliana.* On the other hand, Sanchez-López et al. (2016) determined that the volatiles produced by some microorganisms, such as *Escherichia coli, Penicillum sp.* or *Agrobacterium sp.*, can accelerate flowering in *A. thaliana* plants. Our results agree with some of these results and show that the inoculation with the FO12 strain could accelerate the flowering process in cucumber plants cultured in calcareous soils (Fig. 10).

In conclusion, the ability of the FO12 strain to colonize cucumber roots and induce Fe deficiency responses, and to improve the growth and development of the plants in a calcareous soil, indicates that this fungus could have a great potential as a Fe biofertilizer, but further research is required.

### ***Author contributions statement***

MAA performed experiments and wrote the paper. FJR and PJA helped with the experimental procedures. FJR helped with statistical analysis. MSL conducted NO determination with fluorescence microscopy. PP performed colonization analysis of roots. RP and MJG conducted analysis of gene expression. CL and EA conducted greenhouse experiments. CL, JR and FJR helped with the design of experiments and revised the manuscript. All authors read and approved the manuscript.

## Data Availability

The original contributions presented in the study are included in the article. Further inquiries can be directed to the corresponding author.

## Abbreviations

CLSM: Confocal laser scanning microscopy
dai: Days after inoculation
EMR: Endophytic-mediated resistance
ISR: Induced systemic resistance
TF: Transcription factor
